# The Femoral Tunnel Drilling Angle at 45° Coronal and 45° Sagittal Provided the Lowest Peak Stress and Strain on the Bone Tunnels and Anterior Cruciate Ligament Graft

**DOI:** 10.3389/fbioe.2021.797389

**Published:** 2021-11-26

**Authors:** Rongshan Cheng, Huizhi Wang, Ziang Jiang, Dimitris Dimitriou, Cheng-Kung Cheng, Tsung-Yuan Tsai

**Affiliations:** ^1^ School of Biomedical Engineering & Med-X Research Institute, Shanghai Jiao Tong University, Engineering Research Center of Digital Medicine and Clinical Translation, Ministry of Education, Shanghai, China; ^2^ Shanghai Key Laboratory of Orthopaedic Implants & Clinical Translation R&D Center of 3D Printing Technology, Department of Orthopaedic Surgery, Shanghai Ninth People’s Hospital, Shanghai Jiao Tong University School of Medicine, Shanghai, China; ^3^ Department of Orthopedics Balgrist University Hospital, Forchstrasse, Zürich, Switzerland

**Keywords:** femoral tunnel drilling angle, femoral and tibial tunnel, bone tunnel enlargement, graft failure, anterior cruciate ligament reconstruction, finite element analysis

## Abstract

**Purpose:** The aims of this study were to 1) investigate the effects of femoral drilling angle in coronal and sagittal planes on the stress and strain distribution around the femoral and tibial tunnel entrance and the stress distribution on the graft, following anterior cruciate ligament reconstruction (ACLR), 2) identify the optimal femoral drilling angle to reduce the risk of the tunnel enlargement and graft failure.

**Methods:** A validated three-dimensional (3D) finite element model of a healthy right cadaveric knee was used to simulate an anatomic ACLR with the anteromedial (AM) portal technique. Combined loading of 103.0 N anterior tibial load, 7.5 Nm internal rotation moment, and 6.9 Nm valgus moment during normal human walking at joint flexion of 20° was applied to the ACLR knee models using different tunnel angles (30°/45°/60° and 45°/60° in the coronal and sagittal planes, respectively). The distribution of von Mises stress and strain around the tunnel entrances and the graft was calculated and compared among the different finite element ACLR models with varying femoral drilling angles.

**Results:** With an increasing coronal obliquity drilling angle (30° to 60°), the peak stress and maximum strain on the femoral and tibial tunnel decreased from 30° to 45° and increased from 45° to 60°, respectively. With an increasing sagittal obliquity drilling angle (45° to 60°), the peak stress and the maximum strain on the bone tunnels increased. The lowest peak stress and maximum strain at the ACL tunnels were observed at 45° coronal/45° sagittal drilling angle (7.5 MPa and 7,568.3 μ-strain at the femoral tunnel entrance, and 4.0 MPa and 4,128.7 μ-strain at the tibial tunnel entrance). The lowest peak stress on the ACL graft occurred at 45° coronal/45° sagittal (27.8 MPa) drilling angle.

**Conclusions:** The femoral tunnel drilling angle could affect both the stress and strain distribution on the femoral tunnel, tibial tunnel, and graft. A femoral tunnel drilling angle of 45° coronal/ 45° sagittal demonstrated the lowest peak stress, maximum strain on the femoral and tibial tunnel entrance, and the lowest peak stress on the ACL graft.

## Introduction

Anterior cruciate ligament (ACL) rupture is one of the most common ligamentous injuries of the knee joint (Griffin et al., 2006). ACL reconstruction (ACLR) is the commonly treatment for ACL injuries, with a success rate of up to 90% in knee function at short-term follow-ups (Oiestad et al., 2010). However, long-term follow-up studies have reported several complications following ACLR. Neblung et al. (Nebelung et al., 1998) reported that 72% patients demonstrated enlargement of the femoral tunnel and 38% patients demonstrated enlargement of the tibial tunnel following ACLR, respectively. Xu et al. (Xu et al., 2011) found that the rates of tunnel enlargement were 41% around the femoral side and 35% around the tibial side. Sonnery-Cottet et al. (Sonnery-Cottet et al., 2017) found that 10.77% of ACLR patients with quadrupled hamstring tendon graft and 16.77% with bone-patellar-tendon-bone (BPTB) graft suffered a graft rupture in a 4-years follow-up study. Furthermore, previous studies (Weber et al., 2015; Wang et al., 2020a) reported that tunnel enlargement and graft fatigue failure occurred at the bone tunnel aperture, which might be due to high contact stress or strain between the bone tunnel aperture and the graft. Hence, tunnel enlargement and graft fatigue failure raise relevant concerns following ACLR.

Several studies (Schechtman and Bader, 1997; Jagodzinski et al., 2005; Yao et al., 2014; Srinivas et al., 2016; Srinivas et al., 2016) have investigated the factors that might cause tunnel enlargement and graft failure. Srinivas et al. (Srinivas et al., 2016) found that the enlargement of femoral tunnel and tibial tunnel varied with different methods of fixation. L'Insalata et al. (L'Insalata et al., 1997) showed tunnel enlargement was significantly greater following ACL reconstruction using hamstring (HS) autograft than in those using bone-patellar-tendon-bone (BPTB) autograft. Schechtman et al. (Schechtman and Bader, 1997) reported a linear relationship between the stress and the number of cycles of tendons to fatigue failure. Few studies demonstrated that tunnel orientation might influence tunnel enlargement and graft failure (Jagodzinski et al., 2005; Yao et al., 2014). Specifically, Yao et al. (Yao et al., 2014) quantified the effects of tibial tunnel drill-guide angle on the stress redistribution at the tibial tunnel aperture after ACLR, which potentially contributed to the tibial tunnel widening. A cadaver study (Jagodzinski et al., 2005) reported that the bone tunnel angle might affect the redirecting force between the femoral tunnel and the graft. It may be an essential factor causing tunnel enlargement and graft failure. However, to the best of our knowledge, no study has determined the optimal femoral drilling angle to provide a better mechanical environment for the bone tunnel and the graft to prevent tunnel enlargement and graft failure.

The aims of this study were to 1) investigate the effects of femoral drilling angle in coronal and sagittal planes on the stress and strain distribution around the bone tunnels and the stress distribution of the graft, following anterior cruciate ligament reconstruction (ACLR), 2) to identify the optimal femoral drilling angle to reduce the risk of the tunnel enlargement and ACL graft failure. It was hypothesized that the femoral tunnel drilling angle in coronal and sagittal planes could affect the stress and strain distribution around the femoral and tibial tunnel entrance and the graft.

## Methods

### Finite Element Model of the Knee Joint

**FIGURE 1:**
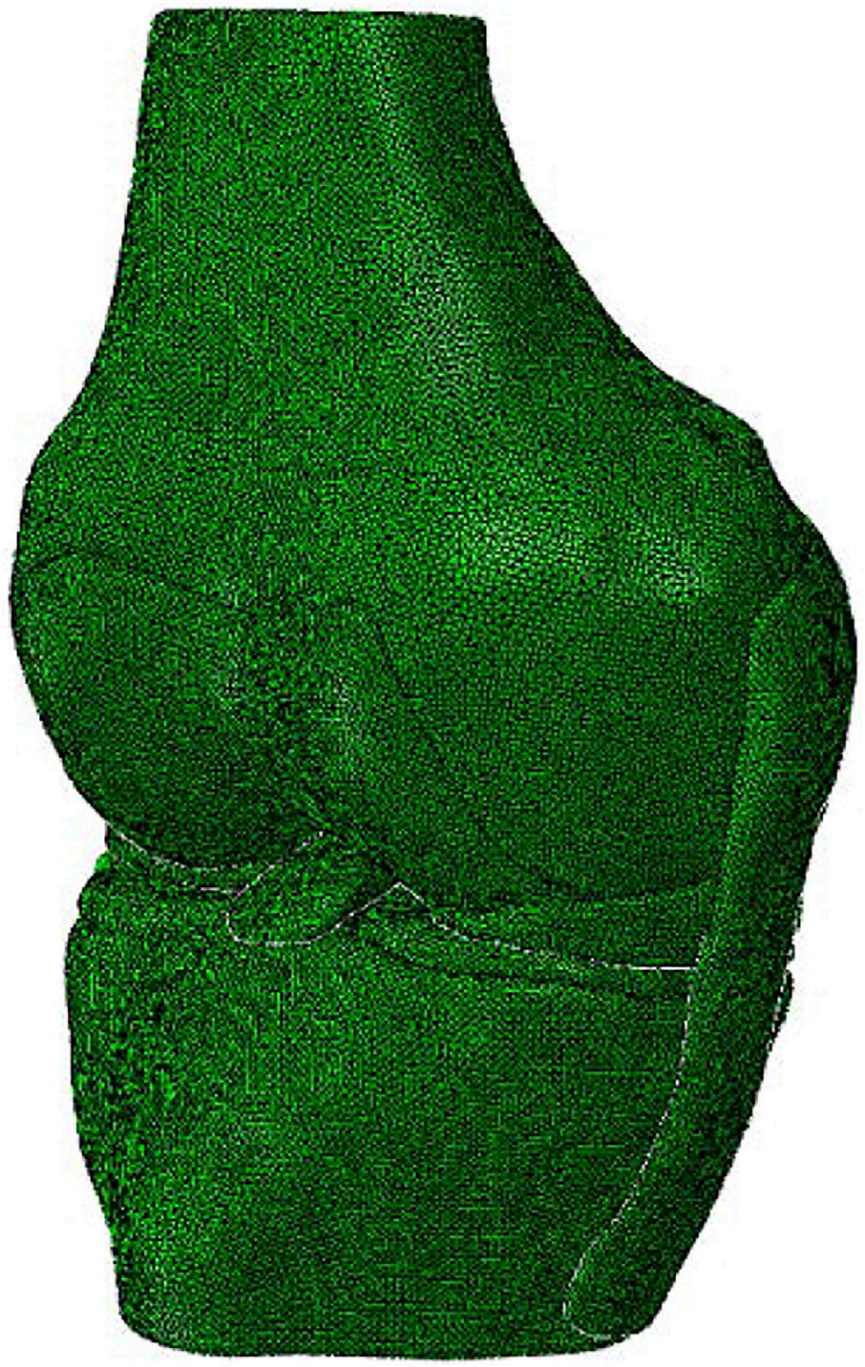
A three dimensional **(3D)** finite element model of the knee joint was reconstructed using Abaqus/CAE 6.14.

A three-dimensional (3D) FE model of a healthy right cadaveric knee (male, 45 years) was reconstructed and validated in a published study using software ABAQUS 6.14 (Simulia Inc., United States) (Wang et al., 2020a; Figure 1). Details about the construction and validation of the knee FE model were shown as follows. The cadaveric knee was imaged using magnetic resonance imaging (MRI) (SIEMENS MAGNETOM Skyra, SIEMENS, Germany) with a 0.2 mm resolution (TE/TR = 26.3 and 53 ms). 3D model of the knee was reconstructed from the MRI images and consisted of ligaments [ACL, posterior cruciate ligament (PCL), medial and lateral collateral ligament (MCL and LCL)], bones, cartilage, and menisci. The mechanical characteristics of the bone and the tissues were defined from the literature (Wang et al., 2020b; Table 1). A pre-strain of 3% was defined for the ACL (Song et al., 2004). A frictionless sliding contact was established among the femoral and tibial cartilage and the meniscus (Wang et al., 2020b). A tie contact was defined between the ligaments and the bone insertions (Wang et al., 2020b). The model was meshed in ABAQUS software using 4-node tetrahedron elements. To optimize the element size of the model, we used a mesh convergence test, whereby the element size was determined until the result for ACL *in-situ* force (under a 2.5 mm translational load at full extension) converged with a calculation difference within 0.5 N (Wang et al., 2020a). The resulting validated element size was 1 mm. Validation of the knee FE model: the differences between the results of robotic testing and the FE model were within 0.1 mm, 1° and 1 N for anterior tibial translation, rotation of valgus, and ACL *in-situ* force, respectively (Wang et al., 2020b) (Table 2).

**TABLE 1:** Material properties of the tissues in the knee model (Wang et al., 2020b).

*Tissue*	*Material type*	*Parameters*
Bone	Isotropic elastic	Young’s modulus = 0.4 GPa, Poisson’s ratio v = 0.33
Cartilage	Isotropic elastic	Young’s Modulus = 5 MPa, Poisson’s ratio v = 0.46
Menisci	Orthotropic elastic	E_θ_ = 125 MPa, E_R_ = E_Z_ = 27.5 MPa, G_θR_ = G_θZ_ = 2 MPa, G_RZ_ = 10.34, V_θR_ = V_θZ_ = 0.1, V_RZ_ = 0.33
ACL	Isotropic hyperelastic	Veronda-Westmann: *α* = 0.3 MPa, *β* = 12.20
PCL	Isotropic hyperelastic	Veronda-Westmann: *α* = 0.18 MPa, *β* = 17.35
MCL and LCL	Isotropic hyperelastic	Mooney-Rivlin: C1 = 30.1 MPa, C2 = − 27.1 MPa

**TABLE 2:** Anterior tibial translation, valgus rotation and internal rotation of tibial, and ACL *in-situ* force obtained from robotic testing and FE model under the loading conditions 1) 134 N anterior tibial load; 2) 10 Nm valgus moment; 3) 10 Nm internal moment at a joint flexion angle of 30° (Wang et al., 2020b).

	134 N Anterior tibial load		10 Nm valgus moment		10 Nm internal moment	
	Anterior tibial translation (mm)	ACL *in-situ* force (N)	Anterior tibial translation (mm)	ACL *in-situ* force (N)	Anterior tibial translation (mm)	ACL *in-situ* force (N)
Experimental (robotic)	5.1	124	5	42	22 ± 3	41 ± 21
Computational (FE)	5.2	123	4	41	19	62

### Simulation of ACLR

**FIGURE 2:**
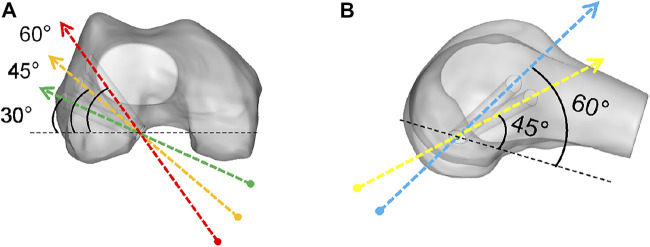
Right knee flexed at 110°, demonstrating the femoral tunnel created by the. anteromedial portal technique at **(A)** a coronal obliquity angle of 30° (green arrow), 45° (orange arrow) and 60° (red arrow), **(B)** a sagittal obliquity angle of 45° (yellow arrow) and 60° (blue arrow), starting at the native femoral ACL center (green circle).

To simulate the anatomic ACLR, we removed the native ACL from the model. The positions of the bone tunnels were determined at 110° knee flexion (Alentorn-Geli et al., 2010). The femoral and tibial tunnels were drilled through the center of ACL insertion sites (Forsythe et al., 2010; Ziegler et al., 2011; Bae et al., 2016). According to the previous studies (Loh et al., 2003; Seon et al., 2011; Warme et al., 2012; Takeda et al., 2013; Zhang et al., 2013; Tomihara et al., 2014), the femoral tunnel was drilled through the anteromedial (AM) portal technique in a coronal obliquity angle of 30°,45°, and 60° and a sagittal obliquity angle of 45° and 60° (Figure 2). The tibial tunnel was created at a tibial angle of 20° in the coronal plane and 60° in the sagittal plane (Alentorn-Geli et al., 2010; Yao et al., 2014). Numbers of studies (Asif et al., 2016; Goyal et al., 2016; Kang et al., 2019; Alomar et al., 2021) reported a graft diameter ≥7 mm was associated with significantly lower ACLR failure rates than a graft diameter <7 mm. A 4-strand hamstring tendon graft was simulated as cylindrical with a diameter of 7 mm (Goyal et al., 2016; Snaebjörnsson et al., 2017; Alomar et al., 2021) and Young’s modulus of 144.8 MPa (Wilson et al., 1999). Titanium endoscrews with a diameter of 7 mm (Young’s modulus / Poisson’s ratio v = 100Gpa and 0.35) (Herbort et al., 2007; Hung et al., 2014; Shen et al., 2018) used in anatomical ACLR were used to secure the graft. The bottom surface of the endoscrews was tied to the ends of the graft, and the outer surface of the endoscrews was connected to the tunnel wall. The screw and the tunnel were concentric.

**FIGURE 3:**
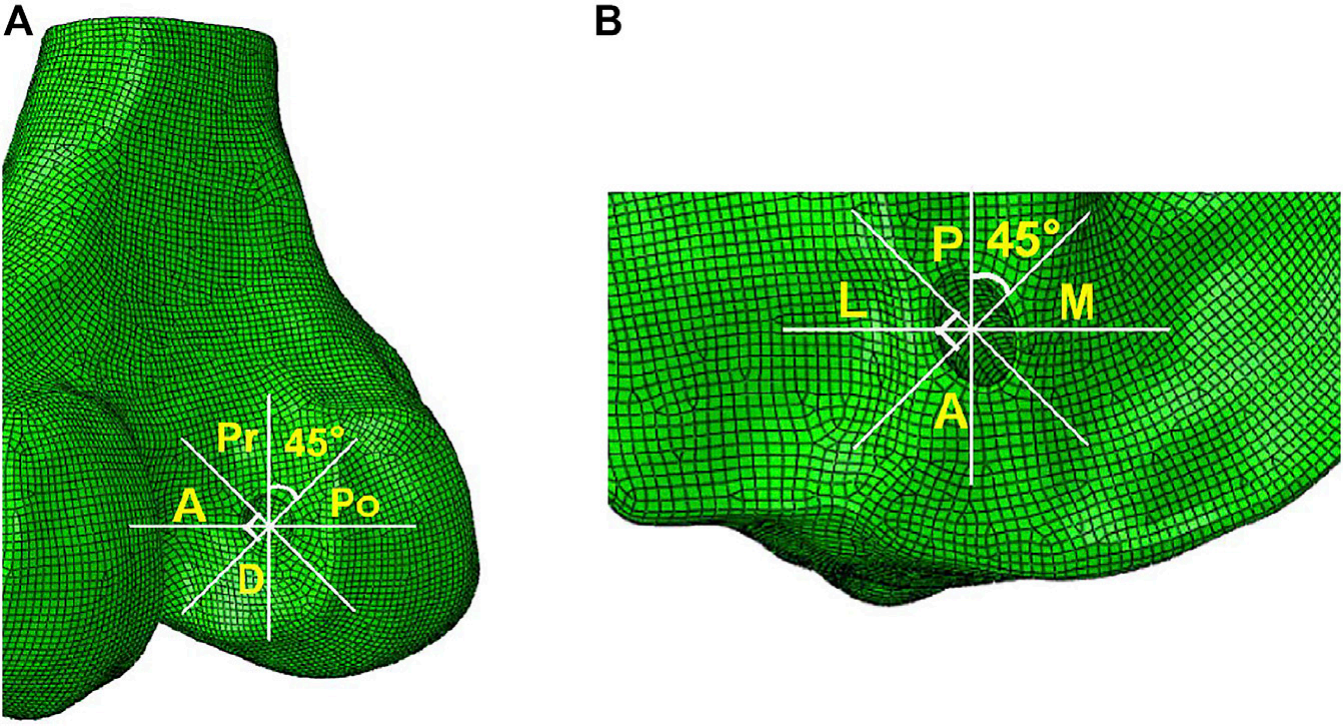
Femoral and tibial tunnel entrances divided into four zones. The two white lines divided the femoral tunnel entrance into four zones: anterior and posterior (A and Po), proximal and distal (Pr and D) zone for the femoral tunnel entrance, and anterior and posterior (A and P), medial and lateral (M and L) zone for the tibial tunnel entrance.

According to the methods described by Wang et al. (Wang et al., 2020a), the entrances of the femoral and tibial tunnel were split into four zones to describe the stress distribution around the bone tunnel entrances: anterior and posterior (A and Po), proximal and distal (Pr and D) zone for the femoral tunnel entrance, and anterior and posterior (A and P), medial and lateral (M and L) zone for the tibial tunnel entrance (Figure 3).

### Loading and Boundary Conditions

The maximum anterior tibial load (103 N, 15% body weight), internal tibial moment (7.5 Nm, 1.1% body weight), and valgus tibial moment (6.9 Nm, 1% body weight) during normal human walking (Kutzner et al., 2010; Wang et al., 2020a; Wang et al., 2020b) were applied to the ACLR model using different tunnel angles at a joint flexion angle of 20° (Wang et al., 2020b). This loading condition represented a worst-case outcome for the ACL during walking (Kutzner et al., 2010). The von Mises stress and strain distribution around the bone tunnel entrances and the graft was calculated and compared among different finite element ACLR models with varying femoral drilling angles in coronal and sagittal planes.

## Results

**FIGURE 4:**
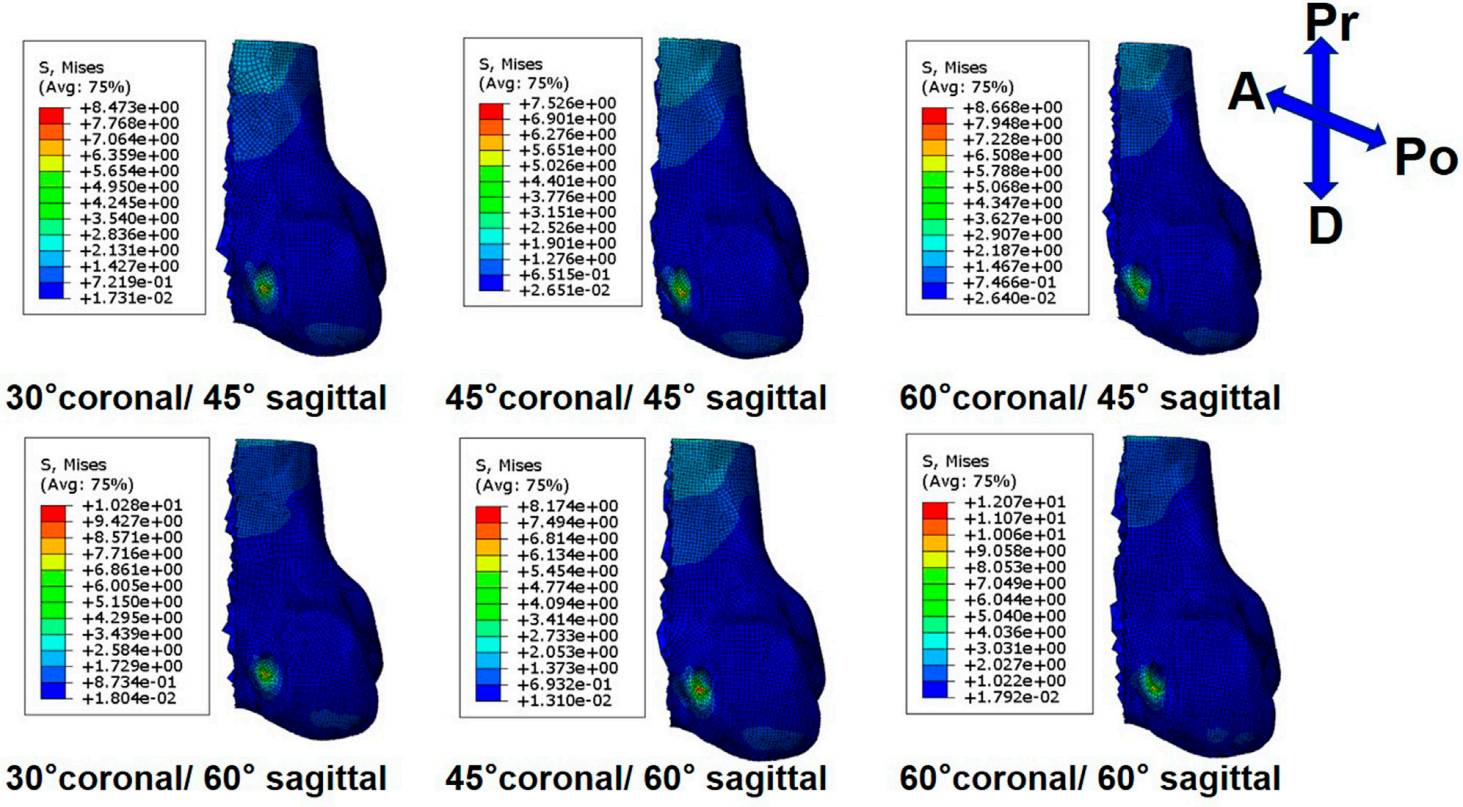
Stress distribution around femoral tunnel entrance following ACLR.

**FIGURE 5:**
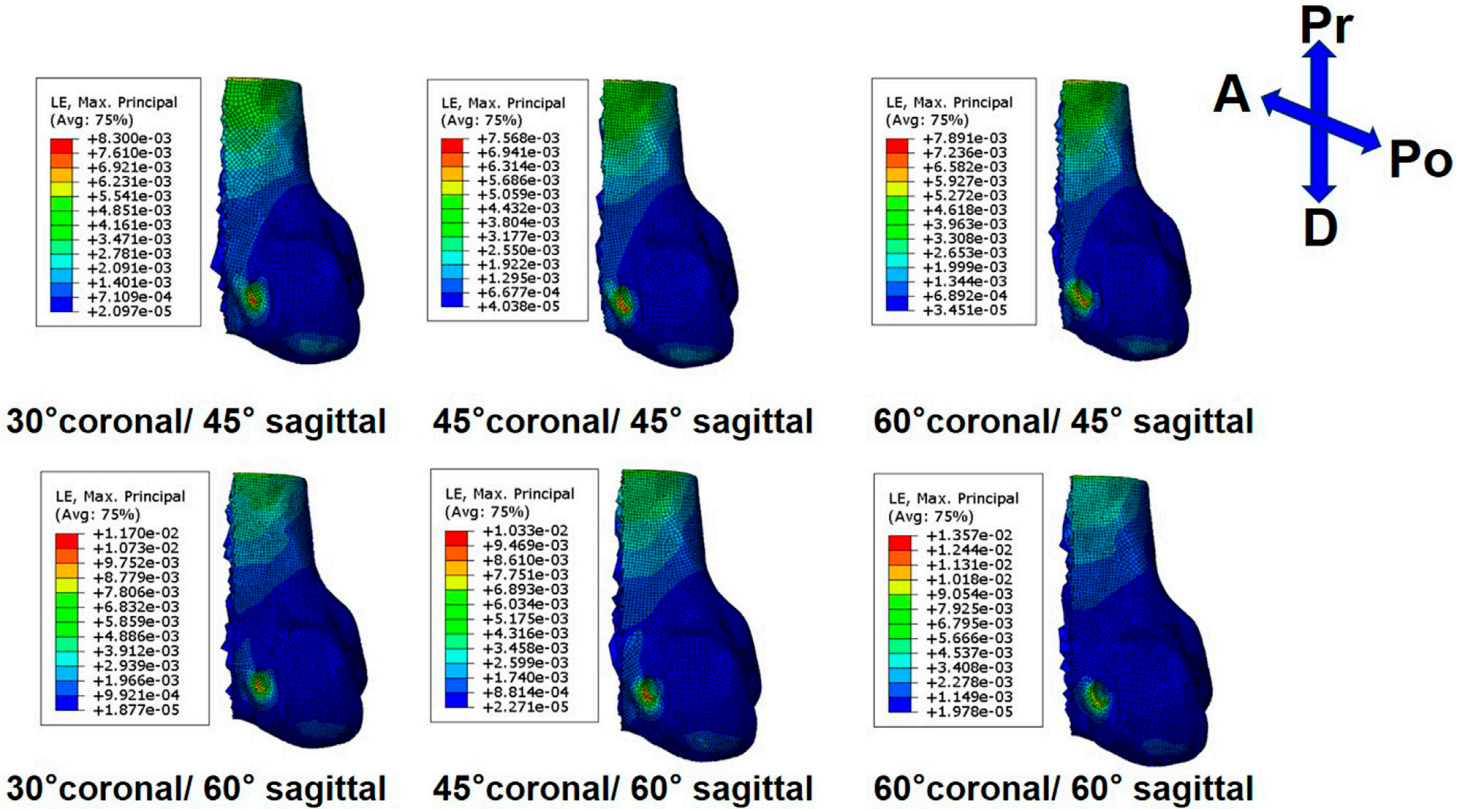
Strain distribution around femoral tunnel entrance following ACLR.

On the femoral side, with an increasing coronal obliquity drilling angle (30° to 60°), the peak stress and maximum strain decreased from 30° to 45° and increased from 45° to 60°, respectively. With an increasing sagittal obliquity drilling angle (45° to 60°), the peak stress and the maximum strain on the bone tunnels increased. The peak stress and the maximum strain in the ACLR knee occurred at the anterior and distal zone of the femoral tunnel entrance (Figures 4, 5) at all angles. The lowest peak stress of the femoral tunnel entrance was 7.5 MPa and occurred in 45° coronal/ 45° sagittal, whereas the highest peak stress with 12.1 MPa occurred in 60° coronal/ 60° sagittal (Table 3). The lowest maximum strain of the femoral tunnel entrance was 7,568.3 μ-strain in 45° coronal/ 45° sagittal, whereas the highest maximum strain with 13,570.8 μ-strain occurred in 60° coronal/ 60° sagittal (Table 4).

**TABLE 3:** Maximum von Mises stress (MPa) at different zones of the tunnel entrances following ACLR by AM portal technique in several coronal and sagittal obliquity angles.

	Femoral tunnel entrance				Tibial tunnel entrance			
	A zone	D zone	Pr zone	Po zone	A zone	M zone	P zone	L zone
ACLR (30° coronal / 45° sagittal)	5.06	8.47	0.55	2.02	0.82	2.24	4.11	0.53
ACLR (45° coronal / 45° sagittal)	5.68	7.53	0.83	1.04	0.75	2.28	3.98	0.44
ACLR (60° coronal / 45° sagittal)	4.98	8.67	1.51	0.84	0.89	1.95	4.01	0.49
ACLR (30° coronal / 60° sagittal)	7.69	10.28	0.54	3.4	1.14	1.64	5.49	0.61
ACLR (45° coronal / 60° sagittal)	4.99	8.17	0.75	1.97	0.67	2.24	4.45	0.51
ACLR (60° coronal / 60° sagittal)	7.36	12.07	1.26	1.44	1.18	2.02	5.48	0.45

**TABLE 4:** Maximum strain (μ-strain) at different zones of the tunnel entrances following ACLR by AM portal technique in several coronal and sagittal obliquity angles.

	Femoral tunnel entrance				Tibial tunnel entrance			
	A zone	D zone	Pr zone	Po zone	A zone	M Zone	P Zone	L Zone
ACLR (30° coronal/45° sagittal)	6896.94	8300.45	1350.07	4217.28	2067.39	3014.44	4429.76	1340.97
ACLR (45° coronal/45° sagittal)	6873.11	7568.32	2070.74	2619.67	1889.14	2893.54	4128.73	759.77
ACLR (60° coronal/45° sagittal)	6941.73	7891.13	3731.91	2142.78	2255.56	3803.60	4571.03	1237.60
ACLR (30° coronal/60° sagittal)	10296.30	11699.20	1611.68	6928.15	2454.41	3433.40	5383.89	1511.62
ACLR (45° coronal/60° sagittal)	9921.03	10327.50	1842.31	4707.57	1715.97	2941.94	4702.43	1287.82
ACLR (60° coronal/60° sagittal)	10454.35	13570.80	3138.72	3678.64	2642.13	3149.21	5424.38	1135.21

**FIGURE 6:**
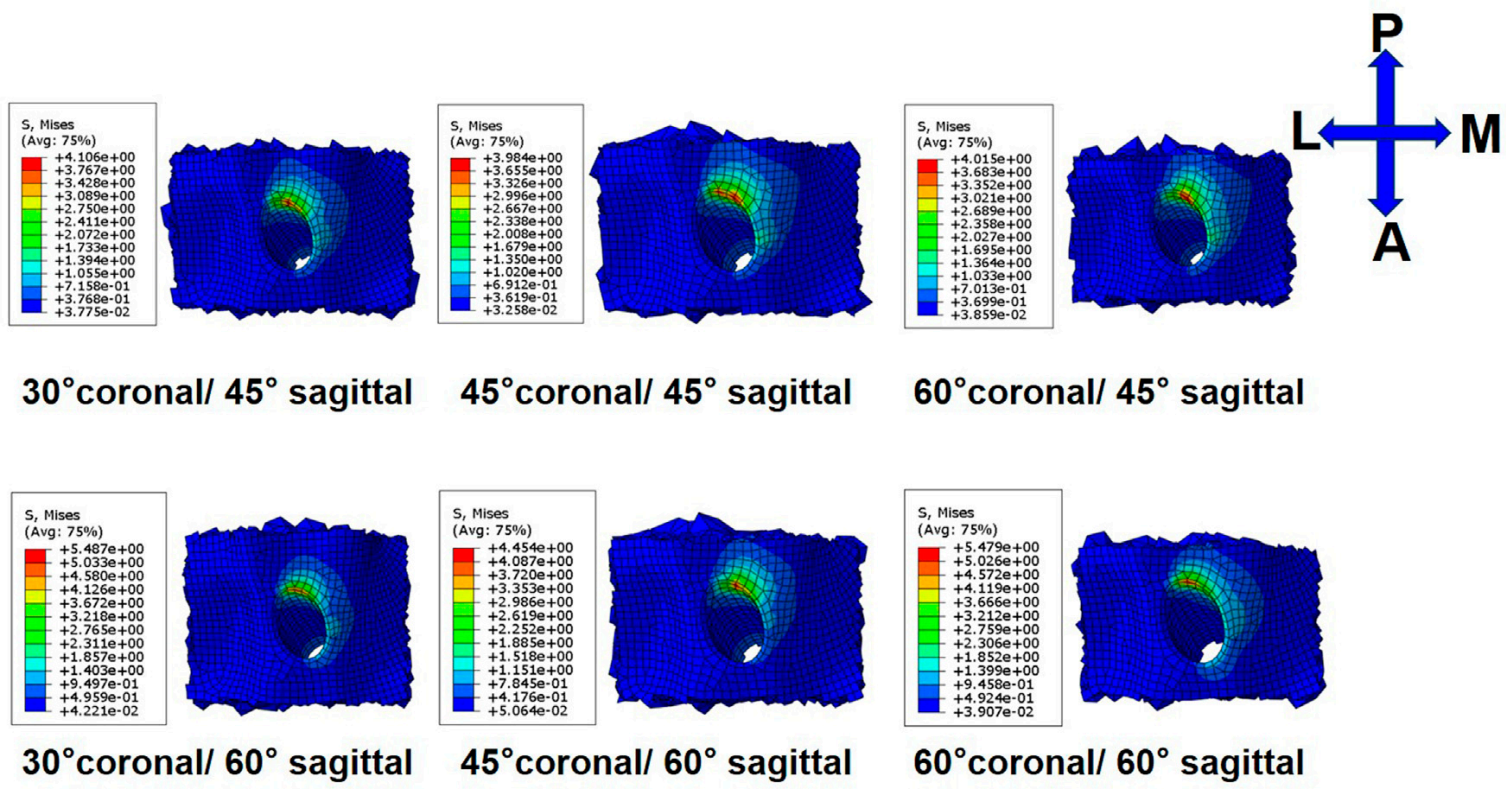
Stress distribution around tibial tunnel entrance following ACLR.

**FIGURE 7:**
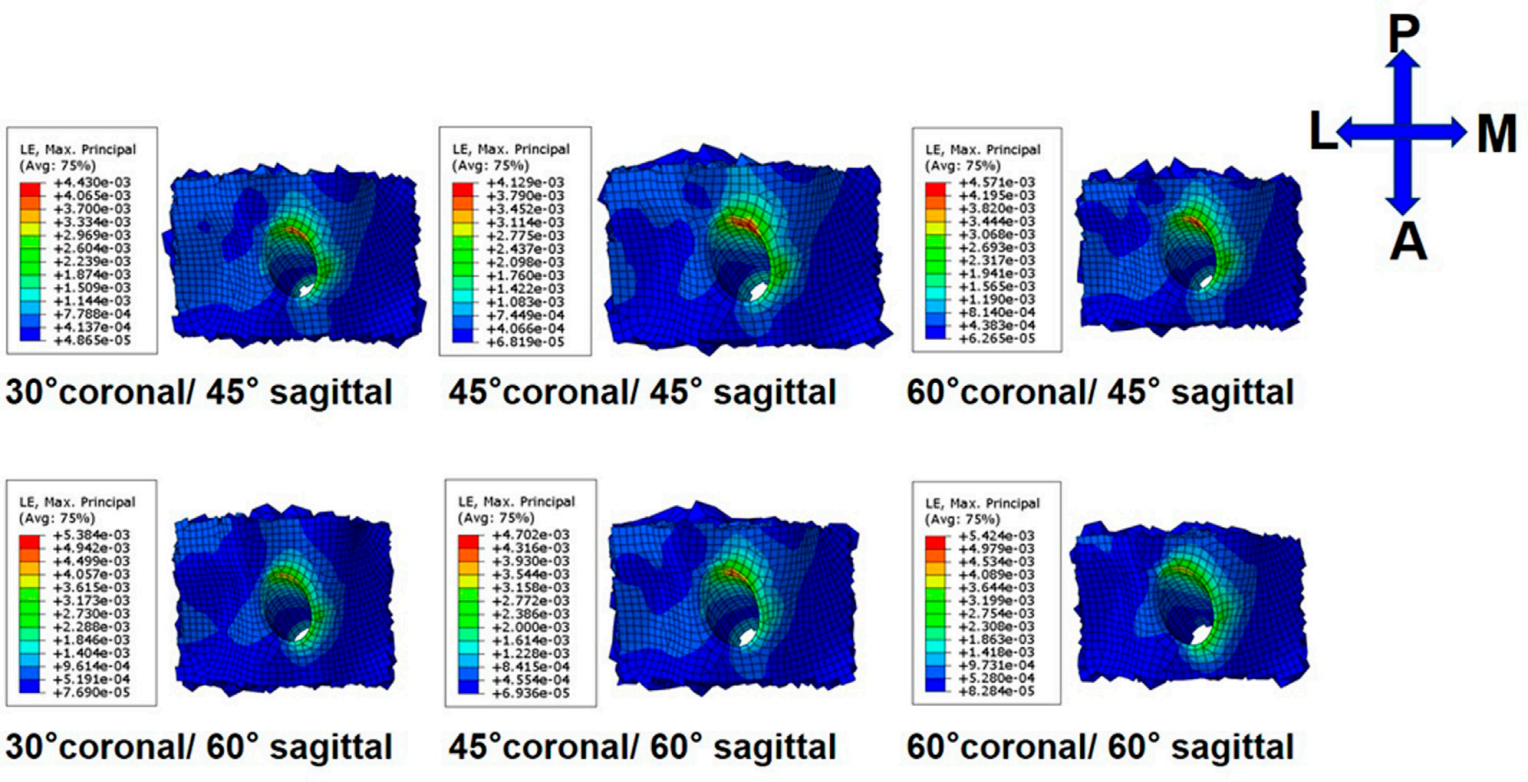
Strain distribution around tibial tunnel entrance following ACLR.

On the tibial side, the trend of the peak stress and maximum strain on the tibial tunnel entrance with coronal and sagittal angle changes was consistent with that on the femoral tunnel entrance. The peak stress and the maximum strain in the ACLR knee occurred at the posterior zone of the tibial tunnel entrance (Figure 6; Figure 7) at all angles. The lowest peak stress of the tibial tunnel entrance was 4.0 MPa and occurred in 45° coronal/ 45° sagittal. In contrast, the highest peak stress with 5.5 MPa occurred in 30° coronal/ 60° sagittal (Table 3). The lowest maximum strain of the tibial tunnel entrance was 4,128.7 μ-strain in 45° coronal/ 45° sagittal, whereas the highest maximum strain with 5,424.4 μ-strain occurred in 60° coronal/ 60° sagittal (Table 4).

**FIGURE 8:**
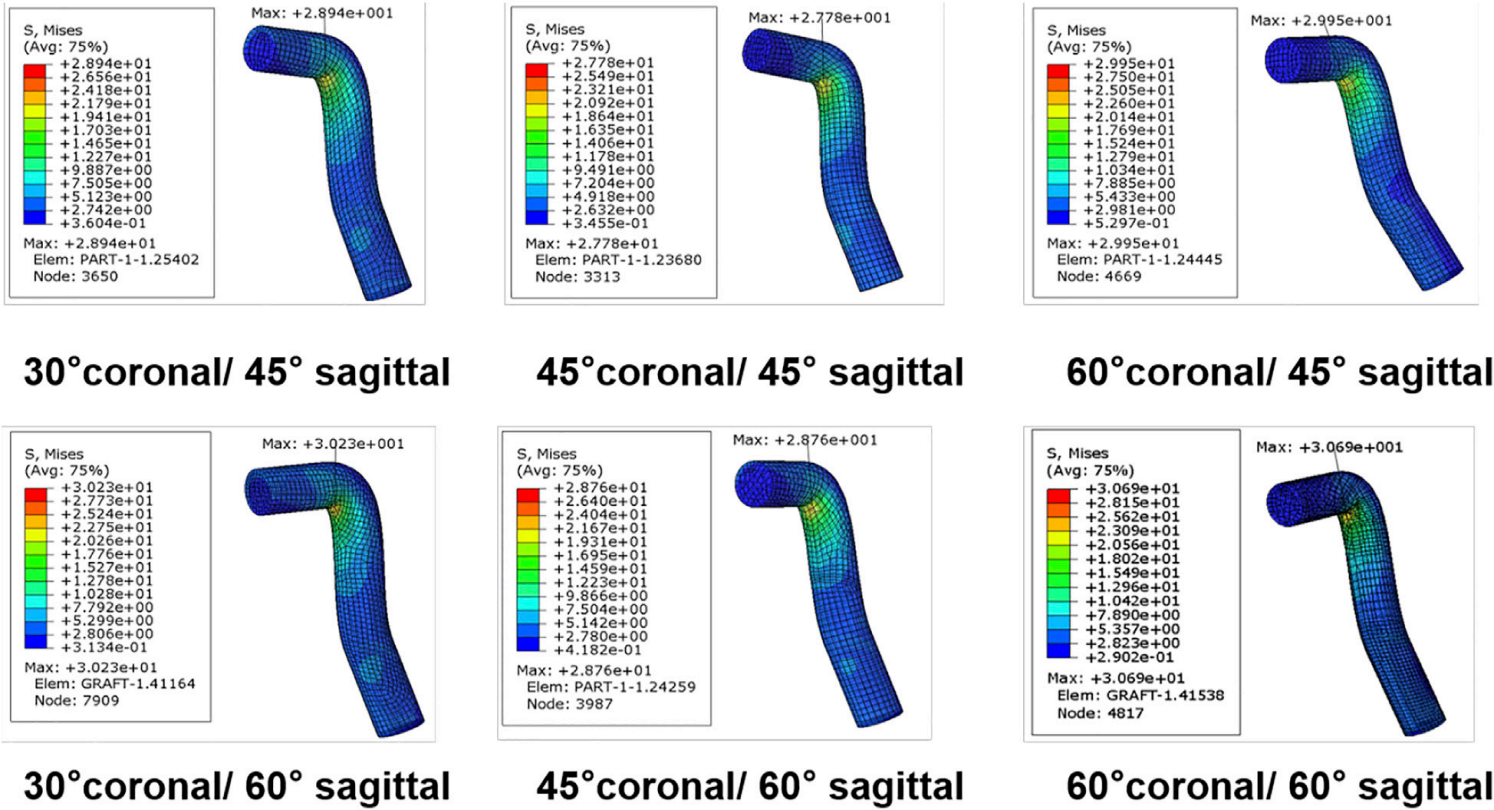
Stress distribution around the ACL graft following ACLR.

The peak stress found on the ACL graft was located close to the entrance of the femoral tunnel (Figure 8). Following ACLR, the highest peak stress on the ACL graft was 30.69 MPa and occurred in 60° coronal/ 60° sagittal. The lowest peak stress on the ACL graft was 27.8 MPa and appeared in 45° coronal/ 45° sagittal.

## Discussion

The most important finding of the present study was that the femoral tunnel drilling angle could affect the stress and strain distribution on both tunnel entrances and the ACL-graft. A femoral tunnel drilling angle of 45° coronal/ 45° sagittal demonstrated the lowest peak stress, maximum strain at both femoral and tibial tunnel entrances, and the lowest peak stress on the ACL graft following ACLR.

The femoral tunnel angle could influence the enlargement of the femoral tunnel (Segawa et al., 2003; Jagodzinski et al., 2005). Jagodzinski et al. (Jagodzinski et al., 2005) found that the bone tunnel angle could affect the force between the bone tunnel and the graft at the entrance of the bone tunnel, which might cause the “bungee effect” and “windshield–wiper effect” of the ACL graft at the tunnel entrances which may be related to the tunnel enlargement. Segawa et al. (Segawa et al., 2003) reported that an acute femoral tunnel angle in the sagittal plane might increase mechanical stress on the margin of the femoral tunnel, resulting in bone tunnel enlargement. Similarly, the present study results demonstrated that the peak stress and the maximum strain at the femoral tunnel entrance increased with an increase in the sagittal obliquity angle. In addition, the peak stress and the maximum strain at the femoral tunnel entrance decreased from 30° to 45° and increased from 45° to 60° in the coronal plane. Previous studies (Wiskott and Belser, 1999; Cheng et al., 2021) showed a critical factor determining the bone loss or formation in response to mechanical loading was the strain value inside the bones. Bone resorption (bone density) was predicted for strain values below the effective strain level (100 μ-strain) or above the low strain level (4,000 μ-strain), whereas bone formation was predicted for strain values between 2000 μ-strain and 4000 μ-strain, and an imbalance between bone resorption and formation was predicted in the strain values range from 100 μ-strain to 2000 μ-strain (Wiskott and Belser, 1999). Our results showed the strain of the distal zone and anterior zone of the femoral tunnel entrance in several coronal and sagittal obliquity angles exceeded 4,000 μ-strain, which could cause an enlargement of the femoral tunnel. In line with our data, a clinical follow-up study (Tachibana et al., 2015) reported that tunnel enlargement occurred in the anterior and distal directions at the femoral tunnel entrance. Therefore, to reduce the femoral tunnel enlargement at the distal zone and anterior zone of the tunnel entrance, a femoral tunnel drilling angle in 45° coronal/ 45° sagittal might be recommended.

The present study is the only available literature to describe the effects of femoral tunnel angle on tibial tunnel enlargement. Our results showed that the trends of change in peak stress and maximum strain at the tibial tunnel entrance in several femoral tunnel drilling angles were consistent with that at the femoral tunnel entrance. Although the trend was consistent, the strain exceeding 4,000 μ-strain only occurred at the posterior zone of the tibial tunnel entrance. Xu et al. (Xu et al., 2011) reported the tunnel enlargement was more evident in the femoral side than in the tibial side, which was in concordance with the current study. The maximum strain at the tibial tunnel entrance was smaller than that at the femoral tunnel entrance. Moreover, Fink et al. (Fink et al., 2001) found that tibial tunnel enlargement was larger in the sagittal plane than that in the coronal plane. This observation was also consistent with our results, as the maximum strain occurred at the posterior zone of the tibial tunnel entrance.

Previous studies (Schechtman and Bader, 1997; Adeeb et al., 2004; Lipps et al., 2013; Purevsuren et al., 2017) reported a high cyclic loading or stress might decrease the number of cycles to cause fatigue failure of ligament and tendon and result in a faster rupture. Our data showed the cyclic stress on the graft following ACLR by AM portal technique in 45° coronal/ 45° sagittal was lowest among several coronal and sagittal obliquity angles, leading to the largest number of cycles to cause a fatigue failure of the graft over time. A cadaver study (Purevsuren et al., 2017) demonstrated the number of cycles for the failure of the ligament and tendon could be predicted by the magnitude of the applied cyclic peak stress [percentage of the ultimate tensile stress (UTS)]. This present study demonstrated that a femoral tunnel drilling angle of 45° coronal/ 45° sagittal showed the lowest peak stress. Therefore, our results showed the femoral tunnel drilling angle at 45° coronal/ 45° sagittal might be optimal to reduce the risk of graft failure. Furthermore, A previous study (Guidoin et al., 2000) showed that most grafts failed at the junction between the femoral tunnel entrance and the graft, which might be caused by the stress concentration. This observation was consistent with our results, as the peak stress on the graft was concentrated at the junction.

The current study should be interpreted in light of its potential limitations. First, a static load was used in this study, and the maximum combined loadings on the ACL during walking were applied to the model. However, different loading conditions may cause different stress environments at the tunnel entrances and the ACL graft. Thus, the influence of different daily activities around the tunnel entrances and the ACL graft may be considered in future studies. Second, several studies (Jagodzinski et al., 2005; Wang et al., 2020a) showed the process leading to tunnel enlargement was thought to be complex and multifactorial. These studies evaluated tunnel enlargement from a biomechanical perspective. In contrast, biological factors may also lead to tunnel enlargement (Yue et al., 2020), which were not considered in this study. In the future, the influence of biological factors on tunnel enlargement and graft fatigue should be quantitatively studied to reinforce our understanding of the mechanism of these complications following ACLR. Third, the present study only focused on the bone tunnel entrances. However, a previous study (Tachibana et al., 2015) showed the tunnel enlargement was more severe at the entrance because the more significant interaction appeared at this location between the tunnel and the graft than that between screw and graft inside the bone tunnel.

## Conclusion

The femoral tunnel drilling angle could affect both the stress and strain distribution on the femoral tunnel, tibial tunnel, and graft. A femoral tunnel drilling angle of 45° coronal/ 45° sagittal demonstrated the lowest peak stress, maximum strain on the femoral and tibial tunnel entrance, and the lowest peak stress on the ACL graft.

## Data Availability

All datasets generated for this study are included in the article/supplementary material.

## References

[B1] Adeeb S. M. Zec M. L. Thornton G. M. Frank C. B. Shrive N. G. (2004). A Novel Application of the Principles of Linear Elastic Fracture Mechanics (LEFM) to the Fatigue Behavior of Tendon Tissue. J. Biomech. Eng. 126 (5), 641–650. 10.1115/1.1800556 15648817

[B2] Alentorn-Geli E. Samitier G. Álvarez P. Steinbacher G. Cugat R. (2010). Anteromedial portal versus Transtibial Drilling Techniques in ACL Reconstruction: a Blinded Cross-Sectional Study at Two- to Five-Year Follow-Up. Int. Orthopaedics (Sicot) 34 (5), 747–754. 10.1007/s00264-010-1000-1 PMC2903180 20401753

[B3] Alomar A. Z. Nasser A. S. B. Kumar A. Kumar M. Das S. Mittal S. (2021). Hamstring Graft Diameter above 7 Mm Has a Lower Risk of Failure Following Anterior Cruciate Ligament Reconstruction. Knee Surg. Sports Traumatol. Arthrosc, 1–10. 10.1007/s00167-021-06503-0 33619635

[B4] Asif N. Ranjan R. Ahmed S. Sabir A. B. Jilani L. Z. Qureshi O. A. (2016). Prediction of Quadruple Hamstring Graft Diameter for Anterior Cruciate Ligament Reconstruction by Anthropometric Measurements. Indian J. Orthop. 50 (1), 49–54. 10.4103/0019-5413.173521 26955176 PMC4759874

[B5] Bae J. Y. Kim G.-H. Seon J. K. Jeon I. (2016). Finite Element Study on the Anatomic Transtibial Technique for Single-Bundle Anterior Cruciate Ligament Reconstruction. Med. Biol. Eng. Comput. 54 (5), 811–820. 10.1007/s11517-015-1372-x 26296801

[B6] Cheng R. Jiang Z. Dimitriou D. Gong W. Tsai T.-Y. (2021). Biomechanical Analysis of Personalised 3D-Printed Clavicle Plates of Different Materials to Treat Midshaft Clavicle Fractures. J. Shanghai Jiaotong Univ. (Sci.) 26 (3), 259–266. 10.1007/s12204-021-2291-7

[B7] Fink C. Zapp M. Benedetto K. P. Hackl W. Hoser C. Rieger M. (2001). Tibial Tunnel Enlargement Following Anterior Cruciate Ligament Reconstruction with Patellar Tendon Autograft. Arthrosc. J. Arthroscopic Relat. Surg. 17 (2), 138–143. 10.1053/jars.2001.21509 11172242

[B8] Forsythe B. Kopf S. Wong A. K. Martins C. A. Anderst W. Tashman S. (2010). The Location of Femoral and Tibial Tunnels in Anatomic Double-Bundle Anterior Cruciate Ligament Reconstruction Analyzed by Three-Dimensional Computed Tomography Models. The J. Bone Jt. Surgery-American Volume 92 (6), 1418–1426. 10.2106/jbjs.I.00654 20516317

[B9] Goyal S. Matias N. Pandey V. Acharya K. (2016). Are Pre-operative Anthropometric Parameters Helpful in Predicting Length and Thickness of Quadrupled Hamstring Graft for ACL Reconstruction in Adults? A Prospective Study and Literature Review. Int. Orthopaedics (Sicot) 40 (1), 173–181. 10.1007/s00264-015-2818-3 26105766

[B10] Griffin L. Y. Albohm M. J. Arendt E. A. Bahr R. Beynnon B. D. DeMaio M. (2006). Understanding and Preventing Noncontact Anterior Cruciate Ligament Injuries. Am. J. Sports Med. 34 (9), 1512–1532. 10.1177/0363546506286866 16905673

[B11] Guidoin M.-F. Marois Y. Bejui J. Poddevin N. King M. W. Guidoin R. (2000). Analysis of Retrieved Polymer Fiber Based Replacements for the ACL. Biomaterials 21 (23), 2461–2474. 10.1016/s0142-9612(00)00114-9 11055294

[B12] Herbort M. Weimann A. Zantop T. Strobel M. Raschke M. Petersen W. (2007). Initial Fixation Strength of a New Hybrid Technique for Femoral ACL Graft Fixation: the Bone Wedge Technique. Arch. Orthop. Trauma Surg. 127 (9), 769–775. 10.1007/s00402-006-0217-3 16937139

[B13] Hung C.-C. Chen W.-C. Yang C.-T. Cheng C.-K. Chen C.-H. Lai Y.-S. (2014). Interference Screw versus Endoscrew Fixation for Anterior Cruciate Ligament Reconstruction: A Biomechanical Comparative Study in Sawbones and Porcine Knees. J. Orthopaedic Translation 2 (2), 82–90. 10.1016/j.jot.2014.02.001

[B14] Jagodzinski M. Foerstemann T. Mall G. Krettek C. Bosch U. Paessler H. H. (2005). Analysis of Forces of ACL Reconstructions at the Tunnel Entrance: Is Tunnel Enlargement a Biomechanical Problem? J. Biomech. 38 (1), 23–31. 10.1016/j.jbiomech.2004.03.021 15519336

[B15] Kang H. Dong C. Wang F. (2019). Small Hamstring Autograft Is Defined by a Cut-Off Diameter of 7 Mm and Not Recommended with Allograft Augmentation in Single-Bundle ACL Reconstruction. Knee Surg. Sports Traumatol. Arthrosc. 27 (11), 3650–3659. 10.1007/s00167-019-05475-6 30919001

[B16] Kutzner I. Heinlein B. Graichen F. Bender A. Rohlmann A. Halder A. (2010). Loading of the Knee Joint during Activities of Daily Living Measured *In Vivo* in Five Subjects. J. Biomech. 43 (11), 2164–2173. 10.1016/j.jbiomech.2010.03.046 20537336

[B17] L'Insalata J. C. Klatt B. Fu F. H. Harner C. D. (1997). Tunnel Expansion Following Anterior Cruciate Ligament Reconstruction: a Comparison of Hamstring and Patellar Tendon Autografts. Knee Surg. Sports Traumatol. Arthrosc. 5 (4), 234–238. 10.1007/s001670050056 9430573

[B18] Lipps D. B. Wojtys E. M. Ashton-Miller J. A. (2013). Anterior Cruciate Ligament Fatigue Failures in Knees Subjected to Repeated Simulated Pivot Landings. Am. J. Sports Med. 41 (5), 1058–1066. 10.1177/0363546513477836 23460331 PMC6388619

[B19] Loh J. C. Fukuda Y. Tsuda E. Steadman R. J. Fu F. H. Woo S. L.-Y. (2003). Knee Stability and Graft Function Following Anterior Cruciate Ligament Reconstruction: Comparison between 11 O'clock and 10 O'clock Femoral Tunnel Placement. Arthrosc. J. Arthroscopic Relat. Surg. 19 (3), 297–304. 10.1053/jars.2003.50084 12627155

[B20] Nebelung W. Becker R. Merkel M. Ropke M. (1998). Bone Tunnel Enlargement after Anterior Cruciate Ligament Reconstruction with Semitendinosus Tendon Using Endobutton Fixation on the Femoral Side. Arthrosc. J. Arthroscopic Relat. Surg. 14 (8), 810–815. 10.1016/s0749-8063(98)70015-5 9848590

[B21] Øiestad B. E. Holm I. Aune A. K. Gunderson R. Myklebust G. Engebretsen L. (2010). Knee Function and Prevalence of Knee Osteoarthritis after Anterior Cruciate Ligament Reconstruction. Am. J. Sports Med. 38 (11), 2201–2210. 10.1177/0363546510373876 20713644

[B22] Purevsuren T. Kwon M. S. Park W. M. Kim K. Jang S. H. Lim Y.-T. (2017). Fatigue Injury Risk in Anterior Cruciate Ligament of Target Side Knee during golf Swing. J. Biomech. 53, 9–14. 10.1016/j.jbiomech.2016.12.007 28118979

[B23] Schechtman H. Bader D. L. (1997). *In Vitro* fatigue of Human Tendons. J. Biomech. 30 (8), 829–835. 10.1016/s0021-9290(97)00033-x 9239568

[B24] Segawa H. Koga Y. Omori G. Sakamoto M. Hara T. (2003). Influence of the Femoral Tunnel Location and Angle on the Contact Pressure in the Femoral Tunnel in Anterior Cruciate Ligament Reconstruction. Am. J. Sports Med. 31 (3), 444–448. 10.1177/03635465030310032001 12750141

[B25] Seon J. K. Park S. J. Lee K. B. Seo H. Y. Kim M. S. Song E. K. (2011). *In Vivo* stability and Clinical Comparison of Anterior Cruciate Ligament Reconstruction Using Low or High Femoral Tunnel Positions. Am. J. Sports Med. 39 (1), 127–133. 10.1177/0363546510377417 20847223

[B26] Shen X. Z. Qu F. Li C. B. Qi W. Lu X. Li H. L. (2018). Comparison between a Novel Human Cortical Bone Screw and Bioabsorbable Interference Screw for Graft Fixation of ACL Reconstruction. Eur. Rev. Med. Pharmacol. Sci. 22 (1 Suppl. l), 111–118. 10.26355/eurrev_201807_15372 30004555

[B27] Snaebjörnsson T. Hamrin Senorski E. Ayeni O. R. Alentorn-Geli E. Krupic F. Norberg F. (2017). Graft Diameter as a Predictor for Revision Anterior Cruciate Ligament Reconstruction and KOOS and EQ-5D Values: A Cohort Study from the Swedish National Knee Ligament Register Based on 2240 Patients. Am. J. Sports Med. 45 (9), 2092–2097. 10.1177/0363546517704177 28460194

[B28] Song Y. Debski R. E. Musahl V. Thomas M. Woo S. L.-Y. (2004). A Three-Dimensional Finite Element Model of the Human Anterior Cruciate Ligament: a Computational Analysis with Experimental Validation. J. Biomech. 37 (3), 383–390. 10.1016/s0021-9290(03)00261-6 14757458

[B29] Sonnery-Cottet B. Saithna A. Cavalier M. Kajetanek C. Temponi E. F. Daggett M. (2017). Anterolateral Ligament Reconstruction Is Associated with Significantly Reduced ACL Graft Rupture Rates at a Minimum Follow-Up of 2 years: A Prospective Comparative Study of 502 Patients from the SANTI Study Group. Am. J. Sports Med. 45 (7), 1547–1557. 10.1177/0363546516686057 28151693

[B30] Srinivas D. K. Kanthila M. Saya R. P. Vidyasagar J. (2016). Femoral and Tibial Tunnel Widening Following Anterior Cruciate Ligament Reconstruction Using Various Modalities of Fixation: A Prospective Observational Study. Jcdr 10 (11), Rc09–rc11. 10.7860/jcdr/2016/22660.8907 PMC5198409 28050456

[B31] Tachibana Y. Mae T. Shino K. Kanamoto T. Sugamoto K. Yoshikawa H. (2015). Morphological Changes in Femoral Tunnels after Anatomic Anterior Cruciate Ligament Reconstruction. Knee Surg. Sports Traumatol. Arthrosc. 23 (12), 3591–3600. 10.1007/s00167-014-3252-6 25160473

[B32] Takeda Y. Iwame T. Takasago T. Kondo K. Goto T. Fujii K. (2013). Comparison of Tunnel Orientation between Transtibial and Anteromedial portal Techniques for Anatomic Double-Bundle Anterior Cruciate Ligament Reconstruction Using 3-dimensional Computed Tomography. Arthrosc. J. Arthroscopic Relat. Surg. 29 (2), 195–204. 10.1016/j.arthro.2012.08.020 23270788

[B33] Tomihara T. Yoshida G. Hara Y. Taniuchi M. Shimada N. (2014). Transparent 3-dimensional CT in Evaluation of Femoral Bone Tunnel Communication after ACL Double-Bundle Reconstruction: Comparison between Outside-In and Transportal Technique. Knee Surg. Sports Traumatol. Arthrosc. 22 (7), 1563–1572. 10.1007/s00167-013-2594-9 23842801

[B34] Wang H. Zhang M. Cheng C. K. (2020b). A Novel protection Liner to Improve Graft-Tunnel Interaction Following Anterior Cruciate Ligament Reconstruction: a Finite Element Analysis. J. Orthop. Surg. Res. 15 (1), 1–10. 10.1186/s13018-020-01755-x 32576207 PMC7310529

[B35] Wang H. Zhang B. Cheng C.-K. (2020a). Stiffness and Shape of the ACL Graft Affects Tunnel Enlargement and Graft Wear. Knee Surg. Sports Traumatol. Arthrosc. 28 (7), 2184–2193. 10.1007/s00167-019-05772-0 31690994

[B36] Warme B. A. Ramme A. J. Willey M. C. Britton C. L. Flint J. H. Amendola A. S. (2012). Reliability of Early Postoperative Radiographic Assessment of Tunnel Placement after Anterior Cruciate Ligament Reconstruction. Arthrosc. J. Arthroscopic Relat. Surg. 28 (7), 942–951. 10.1016/j.arthro.2011.12.010 22381687

[B37] Weber A. E. Delos D. Oltean H. N. Vadasdi K. Cavanaugh J. Potter H. G. (2015). Tibial and Femoral Tunnel Changes after ACL Reconstruction. Am. J. Sports Med. 43 (5), 1147–1156. 10.1177/0363546515570461 25681503

[B38] Wilson T. W. Zafuta M. P. Zobitz M. (1999). A Biomechanical Analysis of Matched Bone-Patellar Tendon-Bone and Double-Looped Semitendinosus and Gracilis Tendon Grafts. Am. J. Sports Med. 27 (2), 202–207. 10.1177/03635465990270021501 10102102

[B39] Wiskott H. W. A. Belser U. C. (1999). Lack of Integration of Smooth Titanium Surfaces: a Working Hypothesis Based on Strains Generated in the Surrounding Bone. Clin. Oral Implants Res. 10 (6), 429–444. 10.1034/j.1600-0501.1999.100601.x 10740452

[B40] Xu Y. Ao Y. Wang J. Yu J. Cui G. (2011). Relation of Tunnel Enlargement and Tunnel Placement after Single-Bundle Anterior Cruciate Ligament Reconstruction. Arthrosc. J. Arthroscopic Relat. Surg. 27 (7), 923–932. 10.1016/j.arthro.2011.02.020 21621372

[B41] Yao J. Wen C. Y. Zhang M. Cheung J. T.-M. Yan C. Chiu K.-Y. (2014). Effect of Tibial Drill-Guide Angle on the Mechanical Environment at Bone Tunnel Aperture after Anatomic Single-Bundle Anterior Cruciate Ligament Reconstruction. Int. Orthopaedics (Sicot) 38 (5), 973–981. 10.1007/s00264-014-2290-5 PMC3997784 24566992

[B42] Yue L. DeFroda S. F. Sullivan K. Garcia D. Owens B. D. (2020). Mechanisms of Bone Tunnel Enlargement Following Anterior Cruciate Ligament Reconstruction. JBJS Rev. 8 (4), e0120. 10.2106/jbjs.rvw.19.00120 32539260

[B43] Zhang C. Xu H. Li X. Wang Y. Zhang Q. Zhu Q. (2013). Oblique Femoral Tunnel or Oblique Graft? A Modified Anteromedial portal Technique to Obtain Vertical Femoral Tunnel and Oblique Graft in Anatomic Anterior Cruciate Ligament Reconstruction. Eur. J. Orthop. Surg. Traumatol. 23 (6), 731–735. 10.1007/s00590-012-1046-4 23412179

[B44] Ziegler C. G. Pietrini S. D. Westerhaus B. D. Anderson C. J. Wijdicks C. A. Johansen S. (2011). Arthroscopically Pertinent Landmarks for Tunnel Positioning in Single-Bundle and Double-Bundle Anterior Cruciate Ligament Reconstructions. Am. J. Sports Med. 39 (4), 743–752. 10.1177/0363546510387511 21173191

